# Management of the Placenta in the Third or Placental Stage of Labor

**Published:** 1888-02

**Authors:** T. Griswold Comstock

**Affiliations:** Master in Obstetrics (Vienna)


### Selections.

### MANAGEMENT OF THE PLACENTA, IN THE THIRD OR PLACENTAL STAGE OF LABOR.

By T. GRISWOLD COMSTOCK, M. A., M. D., Ph. D.,
Master in Obstetrics (Vienna).

When the child is born, the second stage of labor is completed, and the third stage of labor is commenced, and demands the attention of the obstetrician. Having attended over twenty-five hundred cases of labor, I will give my experience :

When the second stage is nearly terminated, and the child is about to be delivered, the practitioner should assist the contractions of the uterus by placing one hand over the region of the fundus, and keep it there until the child is born, and as the uterus goes down, and involution already commences, he should maintain gentle pressure upon the fundus until the placenta is delivered. By means of this simple maneuver, nature will be assisted, and (with the rarest exceptions) the afterbirth will be delivered without any untoward accident. We were formerly taught that the placenta should be delivered in twenty minutes, or means should be taken to deliver it by manual extraction. This is an error, because many cases occur, especially after difficult labors and where forceps have been applied, when the placenta will be delayed for one hour or sometimes even two hours, and not the least harm results. I am now supposing that the practitioner or intelligent nurse remains by the side of the woman with one hand over the fundus, as mentioned above. If, after twenty-five minutes have passed, the parturient patient has no pain, the practitioner may insinuate two fingers into the vagina, guided by the cord, and see if he can find that the afterbirth has come down into the vagina, and seems to be detached. He may then make very gentle traction upon the cord, and, at the same time, press with a little force upon the fundus, and see if it excites any pain. If the woman has no pain, he should wait patiently for from ten to twenty minutes longer, and then may make gentle traction once more, and see if any pain is excited. If no pains result, then let him wait patiently until the hour is up, when he may make careful manipulations over the fundus, and, at the same time, make a little traction upon the cord. He may also ask his patient to blow into one hand and repeat it for several times, and often this will excite a pain. As soon as the pain sets in, let him keep up the abdominal pressure, and make traction upon the cord, so as to assist in the final expulsion of the placenta, until it is out of the vagina, when it should be carefully twisted around so as to bring out with it all the membranes, and make a clean delivery. After the complete expulsion of the placenta and removal of all blood-clots, the practitioner's duties have not ended, for it is better to still keep up pressure for a few minutes, and see that the womb remains contracted, that the involution may be permanent. This plan of treatment, if quietly and persistently carried out, will prove a perfect success in, I might say, almost every case.

Regarding the administration of ergot, I follow the special instruction of Prof. Pajot, of Paris, who says: "*Never give ergot when there is anything in the uterus.*" I think this advice is approved of by the best obstetrists and gynecologists of this country, and for many years I have strictly adhered to it, although I was taught just the opposite by my earlier teachers. After the placenta has been delivered, if you think the involution is not complete, or if you fear that hemorrhage may set up, you may then give a dose of ergot.

I have quoted the high authority of Pajot, and I will now quote Robert Barnes, who says: "*The rule is imperative not to give ergot during the placental stage.*" It is high time that this question should be settled, for we are convinced that ergot is employed as a rule by most physicians in country practice, but it is against reason, science and experience. Our objection to ergot given at this period is, that it may possibly defeat the very object in view. When the uterus contains either the fœtus or the placenta, ergot, if administered, will probably excite irregular and tetanoid contractions, which will lock up the placenta and actually keep it imprisoned within the womb, and render attempts to extract it difficult and even dangerous. The method we have described for removing the placenta was taught us years ago, while a student in Vienna, and is known and recognized in Germany as Crede's method, although it was practiced at a much earlier date by Mauriceau, Busch, R. Barnes, McClintock, Hardy, and others; but, as remarked by Sydney Smith, "it is not the man *who first says a thing* who deserves the credit, *but he who says it so loud and so long that at last he persuades the world it is true*," and for this reason we shall continue to give Crede the credit for this safe and universally successful method for removing the placenta, because he has succeeded in *persuading obstetrical authorities* that it is the best method in the conduct of a labor during the placental stage.

In my recommendation of the removal of the placenta by expression, I do not wish to be understood as opposed to active interference when hemorrhage is present or threatening, or when we have an adherent placenta to deal with. In such an event, of course, it is rational and proper to remove the cause at once, and it may be required to deliver the placenta forthwith by manual interference, in accordance with the rules of obstetrical art (secundum artem).

I will, therefore, close this article by summarizing as follows:

(1) During the last stage of the second period of labor, the obstetrist should press steadily upon the abdomen over the region of the fundus, during each pain, until the child is quite delivered. (2) After the delivery of the child, gentle pressure should be still kept up until the complete expulsion of the afterbirth, and this pressure should be maintained for some minutes, until the practitioner is satisfied that the uterus remains well contracted. (3) This practice, if carried out with requisite skill and patience, will be followed with perfect success, *without the administration of ergot*, and exceptions will occur only very rarely. The aphorism of Blundell, "*Nec temere, nec timide*," is a good one, and is quite as true to-day as it was when asserted by the celebrated authority above quoted. In some instances, the advice of Dr. Cordes, of Geneva, of "armed expectancy," or, perhaps, masterly inactivity may be best, temporarily, but the obstetrist must be the judge, and when nature requires any assistance, it is his duty to act, and act promptly, for the more perfect the physician is in science, the more adroit and skillful will he be in his art; but let him always bear in mind the wise advice of the Roman philosopher, Seneca: "*Omnia ars et scientia, imitatio naturæ est.*"

I may add, finally, that I consider the forceps the child's instrument, and a means to save such pain for the mother, so that, statistically speaking, I have found it requisite to apply them, for the past ten years, about once in thirty cases of labor.

It is always my custom to apply a bandage after the labor is completed. I do not insist that this is always absolutely requisite, but often it is necessary, and, when properly applied by the practitioner himself, it never does any harm.

